# Bullous pemphigoid—What do we know about the most recent therapies?

**DOI:** 10.3389/fmed.2022.1057096

**Published:** 2022-11-03

**Authors:** Faith A. P. Zeng, Dedee F. Murrell

**Affiliations:** ^1^School of Medicine, University of New South Wales, Sydney, NSW, Australia; ^2^Department of Dermatology, St George Hospital, Sydney, NSW, Australia; ^3^The George Institute for Global Health, Sydney, NSW, Australia

**Keywords:** bullous pemphigoid, therapies, eosinophils, eotaxin, interleukin, complement, neonatal Fc receptor, CCR3

## Abstract

**Introduction:**

Bullous pemphigoid (BP) is the most common subtype of autoimmune blistering diseases that primarily affects the elderly and is classically defined by the presence of IgG and/or complement C3 against the BP180 and BP230 hemidesmosome proteins. However, most recent studies have introduced the role of specific eosinophil receptors and chemokine mediators in the pathogenesis of BP which are helpful in identifying new targets for future treatments.

**Areas covered:**

This review will focus on the involvement of eosinophils in BP, including the processes that lead to their recruitment, activation, and regulation. Subsequently, covering new therapeutic options in relation to the role of eosinophils. Eotaxin enhances the recruitment of eosinophils in BP, with CCR3 chemoreceptor that is expressed on eosinophils being identified as a key binding site for eotaxin-1. The pathogenic role of IgE and IL-4 in BP is corroborated by successful treatments with Omalizumab and Dupilumab, respectively. IL-5, IL-17 and IL-23 inhibitors may be effective given their roles in promoting eosinophilia.

**Expert opinion:**

Further research into inhibitors of eotaxin, IL-4, IL-5, IL-17, IL-23, CCR3, and specific complement factors are warranted as preliminary studies have largely identified success in treating BP with these agents. Learning from novel treatments for other IgG-mediated autoimmune diseases may be beneficial.

## Introduction

Bullous pemphigoid (BP) is the most common subtype of autoimmune blistering diseases that primarily affects the elderly and is classically defined by the presence of IgG and/or complement C3 against the BP180 and BP230 hemidesmosome proteins (1, 2). Clinically, BP is a heterogenous disease with a wide spectrum of presentations, but typically manifests as widespread tense blisters and severe pruritus associated with erythematous urticarial plaques (3). Glucocorticoids are the cornerstone of treatment in BP, which although have significantly improved morbidity and mortality, are also associated with severe adverse effects due to the chronicity of the disease and thus treatment (4). Therefore, one of the key principles in the management of BP is to reduce the patient’s cumulative exposure to systemic glucocorticoids with the use of steroid-sparing agents (5). Thus, this review aims to discuss new discoveries in the pathogenesis of BP, focusing on the role of eosinophils, which will serve as a roadmap for future targeted therapies to further reduce the burden of steroid-induced side effects.

## What is already known?

In the early days, a large proportion of experimental research had been dedicated to understanding the role of IgG autoantibodies in the pathogenesis of BP. However, in the past two decades, increasing efforts have been pivoted into exploring the IgE response in BP. Fairley et al., Zone et al., gives evidence for the pathogenicity of IgE in BP from their studies on mouse models (6, 7). These findings formed the basis for the study carried out by Döpp et al. which showed clinically significant correlation between IgE BP180 NC16A-specific antibodies and BP disease severity (8). This is further supported by the increasing pool of case reports/series’ showing that omalizumab, an anti-IgE monoclonal antibody, is a well-tolerated and effective treatment in BP (9–13). Omalizumab appears to be a promising treatment for BP, warranting future randomized controlled trials. Omalizumab has been included in the most recent “Updated S2 K guidelines for the management of BP,” an initiative by the European Academy of Dermatology and Venereology (EADV) (14). A phase 3 study (NCT04128176) of the efficacy of rituximab combined with omalizumab has been registered on the US National Library of Medicine Clinical Trials registry, and will soon begin recruitment of study participants (15).

In 1996, Schmidt and colleagues proved increased levels of IL-4, IL-6, and IL-10 in the blister fluid of BP (16). More recently in 2018, the first case report describing an elderly man in his 80s with recalcitrant BP successfully treated with dupilumab, an IL-4 and IL-13 monoclonal antibody (17). The patient had been given an initial 600 mg loading dose of dupilumab administered subcutaneously, followed by weekly 300 mg subcutaneous injections, which showed resolution of all blisters and undetectable levels of BP180 and BP230 antibodies after 3 months of treatment (17). Since then, there have been further reports of dupilumab as a successful and tolerable treatment for BP (18–21). Dupilumab which was successful in a phase 2 clinical trial of BP and is undergoing phase 3 trial (LIBERTY-BP study) (NCT04206553) at present, has been included in the most recent “Updated S2 K guidelines for the management of BP,” an initiative by the EADV (14, 22). Could there then be a role for anti-IL-6 or anti-IL-10 drugs in the treatment of BP as well?

## The role of eosinophils in bullous pemphigoid and treatment implications

Eosinophils are major effector cells of the human immune system with their impacts likely primarily mediated by their cytoplasmic granules, and are found in a variety of organs including the skin. Whilst it is readily accepted that there is enhanced tissue and serum eosinophil levels in BP, the actual mechanisms in which they cause BP are less understood. One potential pathway described in the literature is that eosinophils stimulate the secretion of matrix metalloproteinase-9 which facilitate the degradation of BP180 and cleavage of the dermo-epidermal junction (DEJ) (23–25).

One study found a positive correlation between tissue eosinophil level and the quantity of inflammatory lesions (26). However, with regards to the relationship between blood eosinophil count and severity of BP measured by the Bullous Pemphigoid Disease Activity Index scores, confounding data exists (10, 27–29). Currently, these are preliminary findings, and larger cohort studies are required for more definitive conclusions to be drawn. In 1974, one of the earliest studies showing correlation between elevated IgE and BP by Arbesman et al., concluded that approximately 70% of patients with disease had elevated IgE (30). However, later studies revealed that the proportion of elevated IgE levels in patients with BP is highly variable, ranging from 22 to 100% (8, 31–39).

This section explores the latest findings on the processes that drive eosinophilia, which can serve as a future roadmap for new therapies in BP. Table 1 summarizes all pharmacological agents mentioned in this review (15, 22, 40–53).

**TABLE 1:** Summary of ongoing/completed trials of novel pharmacologic agents in bullous pemphigoid and other promising agents for consideration (in alphabetical order).

Main therapeutic target(s)	Trade name(s)	Trial phase, masking (NCT number)	Status of trial
**Ongoing/completed trials in bullous pemphigoid**			
Complement C1	Sutimlimab	1, Double (NCT02502903) (40)	Completed
CCR3	(AKST4290)	2, Double (NCT04499235) (41)	Completed
C5aR1	Avdoralimab	2, Open-label (NCT04563923) (42)	Recruiting
Eotaxin-1	Bertilimumab	2, Open-label (NCT02226146) (43)	Completed
IL-5	Mepolizumab	2, Double (NCT01705795) (44)	Completed
IL-5Rα	Benralizumab	3, Double (NCT04612790) (45)	Recruiting
IL-23	Tildrakizumab	1, Open-label (NCT04465292) (46)	Not yet recruiting
IL-4 and IL-13	Dupilumab	3, Double (NCT04206553) (22)	Recruiting
IL-17A and IL-23	Ixekizumab	2, Open-label (NCT03099538) (47)	Completed
IgE (with CD20)	Omalizumab (with Rituximab)	3, Open-label (NCT04128176) (15)	Not yet recruiting
Neonatal Fc receptor (FcRn)	Efgartimod	2/3, Double (NCT05267600) (53)	Recruiting
**To consider future research for potential use in bullous pemphigoid**			
IL-17A	Secukinumab	Evidence from case reports supports its use in bullous pemphigoid (48–50)	
Neonatal Fc receptor (FcRn)	Nipocalimab	Evidence from clinical trials supports their use in IgG-mediated autoimmune disease (51, 52)	

### CCR3 and eotaxin

Chemokines are a specific subtype of chemotactic cytokines that mediate leukocyte trafficking by binding to G-coupled protein receptors (54). To date, it is well described that induction of TH_2_ cells is critical for the production of IgE, and that CCR3 and CCR4 chemokine receptors are preferentially expressed on TH_2_ cells (55, 56). Given that IgE is an important mediator in the pathogenesis of BP, it would be logical to hypothesize that elevated levels of CCR3 and CCR4 ligands are present in the disease—this in alignment with recent reports showing elevated levels of CCR3 and its ligand, eotaxin, in BP (57–59). A phase 2 double-blind clinical trial testing AKST4290, a CCR3 inhibitor, in the treatment of BP (NCT04499235) preliminarily concluded that AKST4290 is efficacious when used in conjunction with mometasone furoate (41).

More specifically, eotaxin-1 and eotaxin-3 are significantly upregulated in the serum and blister fluid of BP patients, and is strongly associated with eosinophil numbers and activation (60, 61). This formed the basis for a phase 2 open-label study of the safety and efficacy of bertilimumab, an anti-eotaxin-1 antibody, in the treatment of patients with newly diagnosed moderate to extensive BP (NCT02226146) (43). The study comprised a treatment period of 4 weeks, with bertilimumab IV infusions on Days 0, 14, and 28, followed by a safety and efficacy follow-up period of approximately 13 weeks. The preliminary results demonstrated that bertilimumab was a safe and efficacious treatment of BP (62). Although promising, larger controlled trials with longer follow-up duration are warranted. Given that chemokines are specific and potent leukocyte chemoattractants, they are suitable for targeted therapies. Therefore, it may also then be beneficial to explore the potential therapeutic effects of anti-eotaxin-3 antibodies and CCR4 inhibitors.

### IL-5

Similar to IL-4, IL-5 is a TH_2_ cell-induced cytokine detected in the blister fluid of patients with BP and characterizes the acute phase of BP (63, 64). IL-5 also functions as a critical cytokine for eosinophilic maturation and functional activity, and is found to increase CCR3 expression in eosinophils—this can explain increased chemotactic activity of eosinophils primed with IL-5 in BP fluid (65–67). However, when put into clinical practice, anti-IL-5 antibodies show controversial results. Simon et al., concluded from a randomized placebo-controlled, double-blind phase 2 pilot study (NCT01705795) that mepolizumab (with steroids), an anti-IL-5 antibody, was not effective in the treatment of BP compared to placebo (with steroids) (44). The primary endpoint for the study was defined as the cumulative rate of relapse-free patients after starting therapy. Although the primary outcome was not significantly different between the mepolizumab and placebo groups, it was found that the former did have markedly lowered blood eosinophil levels nonetheless (44). A small sample size and short follow-up duration were the major limitations in this pilot study. Conversely, a recent case report describes the rapid clinical regression of bullous skin lesions in a patient with BP upon treatment with reslizumab, another anti-IL-5 antibody, allowing tapering of systemic steroid dosage (68). In addition, re-exacerbation of skin lesions were noted upon discontinuation of reslizumab, despite use of maintenance cyclosporin (68). Benralizumab is a humanized monoclonal antibody against IL-5Rα, which has been shown to cause direct apoptosis of eosinophils (69, 70). Currently, recruitment for a randomized clinical trial in Phase 3 is underway to evaluate the use of benralizumab (FJORD study) (NCT04612790) in the treatment of BP (45).

### IL-17 and IL-23

Previously, it was observed in *in vitro* studies that increased release of IL-17 leads to enhanced eosinophilia (71). The relevance of this has been minimally substantiated in three recent case reports on the successful treatment of BP with secukinumab, a humanized monoclonal antibody against IL-17A (48–50). In their case report, Kamata et al. had shown that administration of secukinumab decreased circulating anti-BP180 NC16A antibodies in the patient (50). Interestingly, ixekizumab, another anti-IL-17A (and anti-IL23) monoclonal antibody, was incidentally found to induce clinical remission of BP in a patient with concurrent psoriasis—the latter for which ixekizumab had been indicated (72). However, an open-label phase 2 trial evaluating the use of ixekizumab in BP (NCT03099538) had failed to reach endpoint—primary outcome of cessation of blisters was not achieved in the stipulated timeframe, with small sample size (total of 4 participants enrolled and analyzed) being the major limitation of the study (47).

The relationship between IL-23 and IL-17A is well described in allergic asthma, the paradigm of IgE-mediated disease (73, 74). IL-23 is an important cytokine in promoting and maintaining IL-17, thereby facilitating eosinophilia (75, 76). However, Delli et al. have shown that in eosinophil count was positively related to serum IL-17, but negatively related to IL-23 levels in BP (77). Regardless, given the pathogenic role of IL-23 (and IL-17) in other eosinophil-mediated disease, it would still be beneficial to consider IL-23 inhibitors in BP. With this understanding, an early open-label phase 1 pilot study to evaluate the effects of tildrakizumab, an anti-IL-23 antibody, in the treatment of BP has been approved (NCT04465292) (46).

### Complement

Eosinophils express various receptors, including those of complement such as C3a and C5a which are known to enhance eosinophil recruitment, extravasation, and activation (78, 79). The pathogenic role of complement induced eosinophilia in BP is evidenced by studies in mice showing that passive transfer of complement-fixing autoantibodies primarily against BP180 leads to subepidermal blisters that mimic those of human BP (80). Furthermore, it has been found that animals with complete deficiency in complement C3, C4, C5, and C5aR fail to develop BP lesions upon injection with pathogenic autoantibodies (81–87). Currently, a phase 2 open-label study is underway to test the safety and efficacy of avdoralimab, an antiC5aR1 monoclonal antibody, in the treatment of BP (42).

Convertase enzymes play a key role in complement activation—for instance, C3 convertase is required to cleave C3 into C3a and C3b (88). Additionally, C3 convertase is formed by the products of C1s-catalyzed cleavage of C2 and C4, where optimal C3 convertase activity is only observed with high levels of C1s (89). Although the mechanisms of human complement pathways, and their involvement in the pathogenesis of eosinophilia have been well understood for many years, it was not till recently that research into complement inhibitors in BP have been conducted. More specifically, the first ever study on the safety and tolerability of BIVV009 (Sutimlimab), a humanized IgG4 monoclonal antibody that inhibits C1s, in patients with BP (40). A total 8 patients with BP had completed the study (NCT02502903)—preliminary conclusions were that infusions of 4 weekly 60 mg/kg doses of sutimlimab is effective, with the majority of samples showing absence of C3 deposition at the dermal-epidermal junction; and safe, with mostly mild to moderate side effects such as coryzal symptoms (40). Of note, key limitations of the study include small sample size, short treatment duration, and the lack of overt disease activity in the study population which had prohibited the evaluation of clinical efficacy of sutimlimab in treating BP. Therefore, further research is warranted in determining the true efficacy of sutimlimab in the treatment of BP.

## Learning from novel treatments in other autoimmune diseases

Neonatal Fc Rceptor (FcRn) has been found to play a central role in the homeostasis of IgG (90, 91). The formation of IgG-FcRn complex prevents the degradation of IgG, which allows for the recycling and release of IgG (92). Li et al. demonstrated that FcRn deficient mice were resistant to experimental BP and subtypes of pemphigus, where circulating levels of pathogenic IgG were significantly reduced as compared to the wild type mice (93). Efgartigimod is an IgG1-derived Fc-fragment that binds FcRN thus enhancing degradation of pathogenic IgG. A pilot study by Zakrzewicz et al. has shown that efgartigimod, *in vitro*, can stabilize keratinocyte adhesion in the presence of pathogenic pemphigus antibodies (51). Currently, a phase 3 trial is underway to assess the efficacy and safety of efgartigimod in adults with pemphigus (ADDRESS study) (NCT04598451) (94). Efgartigimod could also then be an effective treatment for other IgG-mediated autoimmune disease like BP. In fact, a seamless 2-part, international phase 2/3 study (BALLAD study) (NCT 05267600) has recently been approved to investigate the efficacy, safety, and tolerability of efgartigimod in BP (53). Author (DM) was involved in the designing of the BALLAD study. Nipocalimab, similar in its mechanism of action to efgartigimod, is currently undergoing a phase 3 trial to assess its efficacy and safety in generalized myasthenia gravis, which is also an IgG-mediated autoimmune disease like BP (NCT04951622) (52). If proven successful and safe, nipocalimab could also then be considered as a therapeutic agent in BP.

## Conclusion

Having more targeted treatments for BP would be beneficial given that existing steroid-sparing immunosuppressants can have devastating side effects due to widespread actions. Therefore, further research into antibodies against eotaxin, IL-4, IL-5, IL-17, IL-23, CCR3, and specific complement factors are warranted as preliminary studies have largely identified success in treating BP with these agents. Additionally, a greater focus on chemokine-targeted therapies (such as anti-eotaxin antibodies) may be favorable given more specific actions than their interleukin cytokine counterparts. Exploring novel treatments for other autoimmune diseases for use in BP, such as FcRn inhibitors would also be beneficial.

## Author contributions

FZ and DM contributed to conceptualization and the writing of the original draft. Both authors contributed to manuscript revision, read, and approved the submitted version.
